# Baltic Group Tick-Borne Encephalitis Virus Phylogeography: Systemic Inconsistency Pattern between Genetic and Geographic Distances

**DOI:** 10.3390/microorganisms8101589

**Published:** 2020-10-15

**Authors:** Andrei A. Deviatkin, Ivan S. Kholodilov, Oxana A. Belova, Sergey V. Bugmyrin, Lubov A. Bespyatova, Anna Y. Ivannikova, Yulia A. Vakulenko, Alexander N. Lukashev, Galina G. Karganova

**Affiliations:** 1 Laboratory of Molecular Biology and Biochemistry, Institute of Molecular Medicine, Sechenov First Moscow State Medical University, 119048 Moscow, Russia; alexander_lukashev@hotmail.com; 2 Laboratory of Biology of Arboviruses, Chumakov Institute of Poliomyelitis and Viral Encephalitides (FSBSI “Chumakov FSC R&D IBP RAS), 108819 Moscow, Russia; ivan-kholodilov@bk.ru (I.S.K.); mikasusha@bk.ru (O.A.B.); strannotut@gmail.com (A.Y.I.); 3 Laboratory for Animal and Plant Parasitology, Institute of Biology of the Karelian Research Centre of the Russian Academy of Sciences (IB KarRC RAS), 185910 Petrozavodsk, Russia; sbugmyr@mail.ru (S.V.B.); gamasina@mail.ru (L.A.B.); 4 Department of Virology, Faculty of Biology, Lomonosov Moscow State University, 119234 Moscow, Russia; vjulia94@gmail.com; 5 Martsinovsky Institute of Medical Parasitology, Tropical and Vector Borne Diseases, Sechenov First Moscow State Medical University, 119435 Moscow, Russia; 6 Department of Organization and Technology of Immunobiological Preparations, Institute for Translational Medicine and Biotechnology, Sechenov First Moscow State Medical University, 119991 Moscow, Russia

**Keywords:** TBEV, Karelia, Baltic TBEV-Sib subgroup, ticks, Baltic, pairwise genetic distance, pairwise geographical distance

## Abstract

Tick-Borne Encephalitis Virus (TBEV) is a dangerous arbovirus widely distributed in Northern Eurasia. The area of this pathogen changes over time. At the beginning of the 2000s, the Ixodes tick populations in Karelia increased. At the same time, the area of *I. persulcatus*, the main vector of the Siberian TBEV subtype, also expanded. Herein, we sequenced 10 viruses isolated from ticks collected in three locations from the Karelia region in 2008–2018. PCR positive samples were passaged in suckling mice or pig embryo kidney cells (PEK). After the second passage in suckling, mice viral RNA was isolated and *E-*gene fragment was sequenced. Viral sequences were expected to be similar or nearly identical. Instead, there was up to a 4.8% difference in nucleotide sequence, comparable with the most diverse viruses belonging to the Baltic subgroup in Siberian TBEV subtype (Baltic TBEV-Sib). To reveal whether this was systemic or incidental, a comprehensive phylogeographical analysis was conducted. Interestingly, viruses within each geographic region demonstrated comparable diversity to the whole Baltic TBEV-Sib. Moreover, Baltic TBEV-Sib has a distribution area limited by three ecological regions. This means that active virus mixing occurs in the vast geographic area forming one common virus pool. The most plausible explanation is the involvement of flying animals in the TBEV spread.

## 1. Introduction

Across arboviruses, TBEV causes the greatest burden to the population of Northern Eurasia. The annual number of registered cases has been about 3500–5000 in the European Union and Russia [1,2]. According to some reports, the actual level of TBEV infection is significantly higher than the number of TBEV cases in humans would suggest. For example, in the 1980s, the TBE vaccination was not available in the Czech Republic. At the same time, 50 out of 434 (11.5%) sera samples collected in this country were positive for anti-TBEV antibodies [3]. This coincides with results obtained in Sweden in 2002: 53 out of 642 (8.3%) patients were TBEV-IgG seropositive, and 96 out of 642 (15.0%) samples had boundary antibody levels [4]. In Ekaterinburg (Russia), sera of 57.8% patients, who did not have a history of TBE disease or vaccination against flaviviruses, had antibody titers in a putative protective titer according to the neutralization test [5]. Direct extrapolation of these values to the whole country is most likely incorrect. On the other hand, it allows for a rough estimate of the TBE lifetime exposure incidence (not less than 1000 per 100,000 population in an endemic region). 

Recently, the TBEV area significantly expanded. Novel foci were revealed in the United Kingdom [6], Denmark [7], Bosnia[8], Netherlands [9], Austria [10], Moldova [11], and the Moscow region (Russia) [12]. In addition, there is an expansion in those countries or regions where the virus had not been observed earlier. For example, the Auvergne–Rhône–Alpes region (France) was not known to be a place of TBEV circulation until three human TBEV cases occurred in 2017–2018 [13]. At the same time, a northward spread of TBEV [14,15,16,17] and TBEV elevation to the higher altitudes [10,18,19] has also been observed.

**Figure 1:**
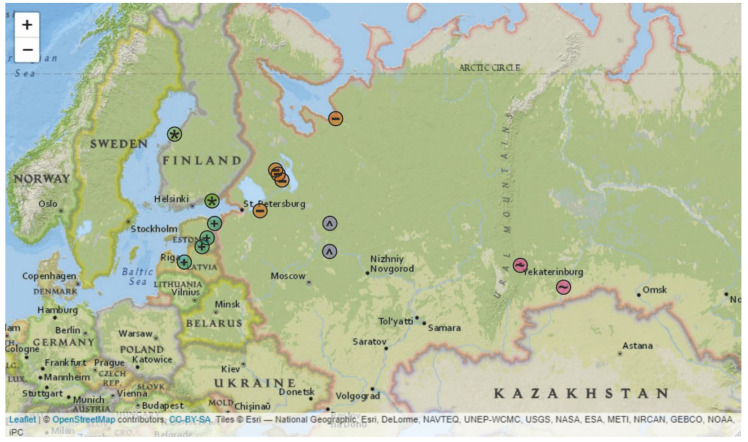
Known distribution of the Baltic subgroup in Siberian TBEV subtype (Baltic TBEV-Sib). Interactive map with labelled markers is available at [30]. Places of tick collection were colour- and symbol-coded according to geographic region: “+” in aquamarinecircle, Estonia and Latvia; “*” in green circle, Finland; “-” in orange circle, Karelia, Arkhangelsk, and Saint-Petersburg regions; “^” in grey circle, Vologda and Yaroslavl regions; and “~” in violet circle, Ekaterinburg and Kurgan regions.

Initially, *TBEV* was subdivided into three major subtypes based on its distribution: European, Siberian, and Far-Eastern subtypes [20]. In general, the distribution of viruses corresponded to the nominal name, although there were many exceptions [21]. According to phylogenetical analysis, the Siberian subtype (TBEV-Sib) consists of several TBEV subgroups [8]. In the 2000s, it became clear that TBEV-Sib viruses were common in the Baltic sea region (Finland, Estonia, Latvia) [22,23,24,25]. These viruses formed a distinct phylogenetic subgroup in Siberian subtype that was called Baltic according to the known geographical distribution. In the late 2000s, representatives of the Baltic subgroup were revealed in the Yaroslavl, Saint-Petersburg, Vologda, Karelia, Ekaterinburg, and Kurgan regions (Russia) [26,27,28,29] (Figure 1). The Karelia, Saint-Petersburg, Yaroslavl, and Vologda regions are in the northern part of European Russia. The Ekaterinburg region is adjacent to the Middle Ural Mountains, whereas the Kurgan region is about 600 km to the east of the Southern Ural Mountains. 

*Ixodes persulcatus* is the main tick vector species known to transmit TBEV-Sib [31]. Karelia is the region where human TBE cases have been reported since 1957 [32]. In the 1950s, the main areas of distribution of *I. persulcatus* and *I.ricinus* in Karelia were described [33]. *I. persulcatus* was abundantly distributed only in the central and eastern regions of Karelia, and its western border of distribution runs along a conventional line between the points N63°15′E33°15′ and N61°15′E31°55′ [34]. Studies in the 2000s revealed a significant increase in the distribution of *I. persulcatus* in Karelia. In addition, a massive increase in the Ixodes tick population was noted in Karelia, in parallel with an increase of TBE morbidity [35,36]. Since 2004, *I. persulcatus* has been recorded in Finland [22]. Currently, *I. persulcatus* has become a widespread species in Finland [37,38]. Moreover, the geographic range of I. persulcatus has recently expanded to northern Sweden [17]. A decade ago, the first TBEV sequences from ticks collected in Petrozavodsk revealed the Siberian TBEV subtype in the southern part of Karelia [28]. Herein, we sequenced one virus from ticks collected in Petrozavodsk, eight viruses from ticks collected from the village of Gomselga (37 km to the north from the capital of Karelia region—Petrozavodsk), and one virus from ticks collected from the village of Pedaselga (33 km to the south of Petrozavodsk). We hypothesized that the viruses from the collected ticks were descendants of one common ancestor that has been recently been introduced in that territory. This hypothesis was proven incorrect upon sequence analysis. Eight viruses from Gomselga differed by up to 4.8% nucleotide sequence. Such diversity is comparable with that within the whole Baltic TBEV-Sib subgroup. To determine whether this was a systemic situation, a comprehensive phylogenetic analysis for Baltic TBEV-Sib subgroup was conducted.

## 2. Materials and Methods 

### 2.1. Tick Collection

Adult questing ticks were collected during field visits to southern Karelia (Russia) in May–June 2006–2018 by flagging (0.7 × 1.1 m) from vegetation. The ticks were kept in moist bandages wrapped in foil or plastic bags and stored at + 4 °C before identification. Taxonomic identification of ticks was done using a binocular microscope (16×) following the recommendations [39].

### 2.2. TBEV Isolation and Sequencing

TBEV was isolated and sequenced as previously described [40]. Briefly, tick suspensions were tested by RT-PCR for the presence of TBEV RNA. Pig embryo kidney (PEK) cells were infected with PCR-positive samples. Two-day-old ICR mice (FSBSI Scientific Center of Biomedical Technologies of Federal Medical Biological Agency, "Stolbovaya" branch, Moscow Oblast, Russian Federation) were injected intracerebrally with 10 μl of virus-containing fluid (infected cell culture supernate, tick suspension). After second passage, viral RNA were isolated from the 10% suspension of suckling mice brain with TRI Reagent LS (Sigma-Aldrich, St. Louis, MO, USA) according to the manufacturer’s protocol. Reverse transcription was performed with virus-specific primers (Kgg30, Kgg32). Viral genomic cDNA was amplified by PCR using TBEV-specific primers Kgg 35, Kgg26, Kgg 16, and Kgg30 [40]. Sequencing was carried out in both directions directly from PCR-amplified DNA on the ABI PRISM 3730 (Applied Biosystems, Waltham, MA, USA) sequencer using ABI PRISM® BigDye™ Terminator v. 3.1. Genomic sequences were assembled using SeqMan software (DNAstar, Madison, WI, USA).

### 2.3. Bioinformatical Analysis

Phylogenetic data were processed as previously described [21] with some modifications. Briefly, all available TBEV sequences represented in GenBank as of March 2020 aligning with genome positions 1150–2200 in the reference sequence #NC_001672 were selected (*n* = 953). For further analysis, Maximum likelihood (ML) phylogenetic inference was performed using IQ-TREE [41]. Then Baltic TBEV-Sib sequences (*n* = 72) were extracted from the whole dataset. Viral sequences from Gomselga (*n* = 8) and Pedaselga (*n* = 1) were manually added to this dataset. The final alignment consisted of 71 sequences, each 1054 nucleotides long.

**Figure 2:**
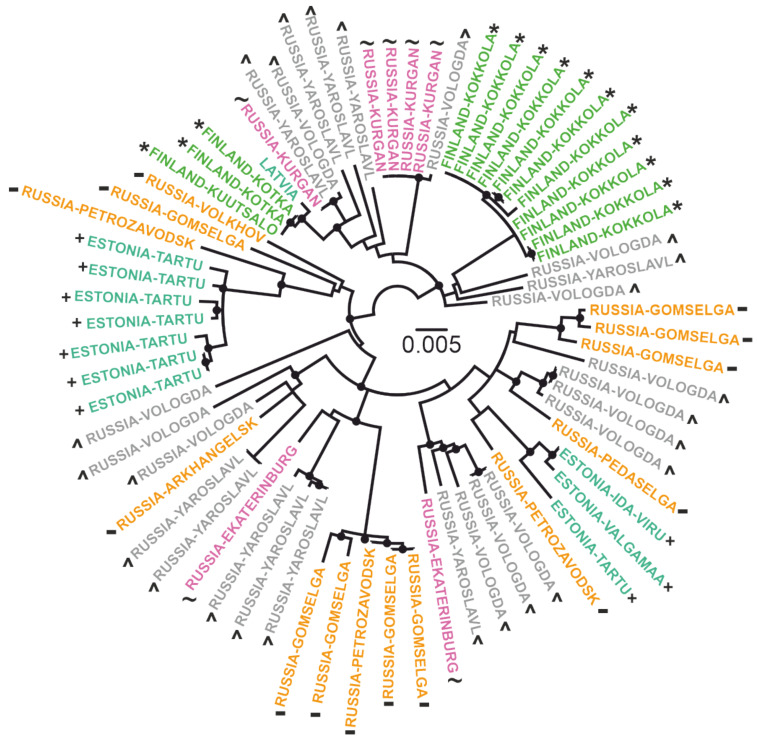
Maximum likelihood tree for Baltic TBEV-Sib (1054 nt). Black circles indicate nodes that were supported by UFBoot values over 95% [43]. Scale bar and branch lengths are the expected number of substitutions per site [44]. Countries or country regions of virus sampling were grouped into five color- and symbol-coded geographical regions: aquamarine, “+” — Estonia and Latvia; green “*”— Finland; orange, “-”—Karelia, Arkhangelsk, and Saint-Petersburg regions; grey, “^” — Vologda and Yaroslavl regions; and violet, “~”—Ekaterinburg and Kurgan regions.

A map with locations of tick collection (Figure 1) was generated in R environment. An interactive map with labelled markers is available at [30]. The ML tree (Figure 2) was constructed using IQ-TREE [41]. The best-fit model was automatically chosen using ModelFinder [42] implemented in IQ-TREE package (v. 1.6.1) according to the Bayesian information criterion. Ultrafast bootstrap (BB) approximation (1000 replicates) was chosen to assess statistical robustness for internal branching order in the phylogeny [43]. Clades with support more than 95% were suggested to be reliable.

**Figure 3:**
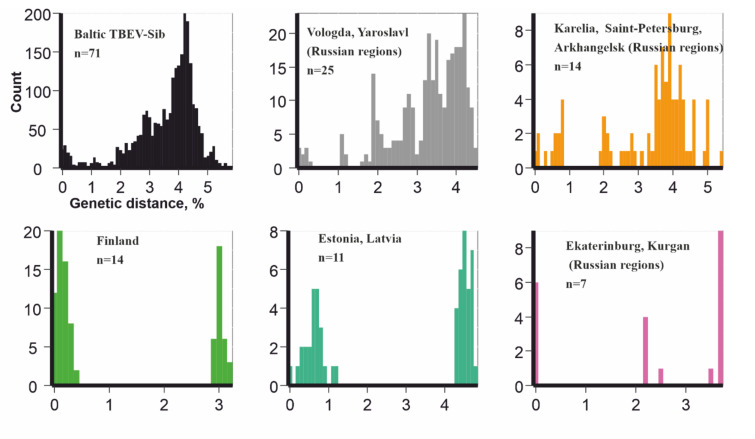
Pairwise genetic distances for all Baltic TBEV-Sib subgroup representatives (*n* = 71); Baltic TBEV-Sib from the Vologda and Yaroslavl regions (*n* = 25); Baltic TBEV-Sib from Karelia, Saint-Petersburg and Arkhangelsk regions (*n* = 14); Baltic TBEV-Sib from Finland (*n* = 14); Baltic TBEV-Sib from Estonia and Latvia (*n* = 11); and Baltic TBEV-Sib from the Ekaterinburg and Kurgan regions (*n* = 7).

**Figure 4:**
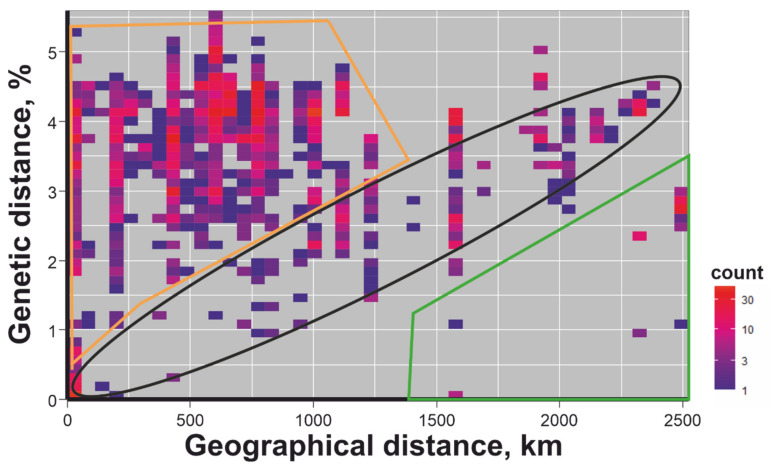
Heatmap of concordance between genetic and geographical pairwise distances for Baltic TBEV. Colour lines indicate suggestive virus spread events discussed in the text.

Pairwise genetic distances distribution (Figure 3) were calculated in R environment. Genetic and geographic distance concordances (Figure 4) were visualized in R environment. 

## 3. Results

There were 10 total viruses sequenced in this study (accession numbers #MT424736-MT424744, MT889225). The 1054 nt fragment of *E-*gene was chosen as traditionally the most represented part of the genome for Baltic TBEV-Sib subgroup for phylogenetic analysis. In order to clarify the locations of tick collection, all of the Baltic TBEV-Sib subgroup was artificially subdivided into five geographical regions: 1) Estonia and Latvia (*n* = 11); 2) Finland (*n* = 14); 3) Saint-Petersburg, Karelia, and Arkhangelsk regions (*n* = 14); 4) Vologda and Yaroslavl regions (*n* = 25); and 5) Ekaterinburg and Kurgan regions (*n* = 7) (Figure 1 and Figure 2).

The phylogenetic analysis found a lack of geographic pattern in phylogenetic grouping, suggesting multiple long-distance transfers. Phylogenetically close viruses possessing common well-supported nodes were isolated from ticks collected in geographically distant regions. For example, four viruses from the Kurgan region (#FJ214128-FJ214131) were nearly identical to TBEV from the Vologda region (#FJ214153), and 1053 out of 1054 nucleotides coincided. At the same time, viruses from one location could be phylogenetically divergent. For example, viruses from the village of Gomselga formed three divergent clades in the phylogenetic tree. In order to compare genetic diversity in five separate geographic regions and the whole Baltic TBEV-Sib subgroup, we calculated the distribution of pairwise distances for these six datasets (Figure 3). Strikingly, all datasets showed comparable genetic diversity. The most diverse pair of viruses, #MT424736 from Gomselga (Russia) and #DQ451293 from Kokkola (Finland), differed by 5.6% in nucleotides. At the same time, #Karl12-T16353 from Petrozavodsk (Russia) and #Karl18-T27106 from Gomselga (37 km to the north from Petrozavodsk) differed by 5.2%. Moreover, #Karl08-T3467 and #MT424737, both found in Gomselga, differed by 4.5% in nucleotides. Such diversity could be formed after hundreds of years of evolution within each location. However, this contradicts the presence of nearly identical viruses isolated in geographically distant locations. Thus, it is most likely that there were several independent (re)introductions of TBEV into Karelia, as well as movements between other areas of the Baltic group. 

To further analyze the phylogeographic patterns of TBEV evolution, we visualised the dependence between pairwise genetic and geographical distances (Figure 4). Every dot on this heatmap plot corresponds to the pair of viruses. The y-axis indicates the percentage of different nucleotides in the studied *E-*gene fragment (1054 nt) between two viruses, whereas the distance (in km) between places of host collection for this pair are shown on the x-axis. The density of occurrence is indicated by colour (legend to Figure 4).

In case of the gradual spread of the virus, we would expect the correlation between genetic and geographic distances that may lead to a linear trend (dots in the black ellipse in Figure 4). Few dots (indicated by green line in Figure 4) represent genetically close viruses isolated at geographically distant locations. Such dots are traces of transfers over a distance of more than 1500 km. Viruses with near-identical or very similar sequences collected in regions separated by thousands of kilometers were likely recently introduced into novel territories. Interestingly, a significantly larger number of virus pairs (selected by orange lines in Figure 4) were genetically diverse but geographically close. As genetic distances of TBEV within and between the five regions are comparable, it is mosl plausible that these dots represent common mixing of viruses between distant regions rather than extensive diversification within a region. It should also be noted that a smaller part of the points in Figure 4 fit into a linear trend, but this could well be a co-incidence. Therefore, Baltic TBEV-Sib spread rather by long-distance jumps than gradually.

## 4. Discussion

Highly (and about equally) diversified Baltic TBEV-Sib was found at five distinct regions of Eurasia. A common, or systematic, presence of diverse viruses at one location could be explained by long-distance TBEV transfers. This may be a result of virus diversification at each location, or the consequence of multiple long-distance tick migrations. The latter hypothesis was favoured by the balance of intra- and inter-location genetic distances and a direct phylogenetic evidence of multiple long-distance transfers. Tick long-distance transfers can only be explained by the assistance of humans (anthropogenic factor) or other animals (zoonotic factor). All known regions where Baltic TBEV was detected belong to the Sarmatic mixed forest, Scandinavian and Russian taiga, and Urals mountain tundra and taiga according to World Wildlife Fund ecoregions nomenclature. Russian regions between Ekaterinburg and Yaroslavl (e.g. Kirov or Udmurtia regions) belong to the same ecological zones and are TBEV endemic territories. For example, 36 out of 160 (22,5%) ticks collected in Kirov region in 2016 were TBEV-positive [45]. Unfortunately, there were just two sequences (out of 976 *E-*gene fragment sequences) from these territories that are publicly available. Both viruses were representatives of the Sib2 TBEV subgroup [21] (prototype strain Zausaev) collected in the Kirov region. In other words, the current study is limited by fragmentary knowledge of the real Baltic TBEV-Sib distribution. 

In addition to the designated regions, the major Sib TBEV vector, *I.persulcatus,* was present in Siberia and the Far-East. If infected Baltic TBEV-Sib ticks were accidentally transferred by humans, the systematic diversity patterns would not be observed. An incidental transfer (or even several transfers) should lead to a random pattern where diversity in all regions would not be comparable. Moreover, in the case of anthropogenic spread, Baltic TBEV-Sib should be detected in other ecological regions where *I. persulcatus* persists—the distribution area of this tick species is significantly wider than the known distribution area of Baltic TBEV-Sib. More than half of the known TBEV sequences are from the Novosibirsk, Irkutsk, and Vladivostok regions. The diversity of TBEV in these regions is very well studied in terms of the number of sequenced viruses, and these cities are centers of transport networks and human activity in the region. Nevertheless, none of around 500 viruses from these regions belonged to Baltic TBEV-Sib. Thus, current evidence indicates that Baltic TBEV-Sib was absent in other ecoregions differing from the mixed forest and taiga in Northeastern Europe and in the boundaries of the Ural mountains. Baltic TBEV-Sib distribution strongly correlated with these zones. Such correlation may be caused by the participation of additional animals in the life cycle of Baltic TBEV-Sib, in which the areal is mainly restricted by these ecoregions. If the virus can replicate in this animal, then it should be an additional TBEV dissemination vector. Otherwise, animals can disseminate TBE indirectly via tick transportation. 

The most plausible explanation of the active mixing of infected ticks is the involvement of flying animals in the life cycle of the TBEV or the ticks. 

Assuming that:

- The known Baltic TBEV-Sib distribution area has borders coinciding with the borders of mixed forest and taiga in Northeastern Europe and the boundaries of Ural mountains;
- *I. persulcatus,* the main vector for the Siberian TBEV subtype, is spread in the territories from Finland and Estonia in the west to Japan in the east [46];
- Baltic TBEV-Sib was not found in Siberia and the Far-East, where TBEV diversity has been extensively explored;
- Baltic TBEV-Sib is a well-mixed population of viruses with comparable diversity in every region.

We may conclude that some flying animal capable of carrying ticks is a necessary missing link for Baltic TBEV-Sib circulation. According to some estimates, the most recent common ancestor of that subgroup existed just hundreds of years ago [21]. If Baltic TBEV-Sib could persist without the involvement of migrating animals, most possibly there would have been traces of (re)introduction into Siberia that currently are not observed. Moreover, in that case, the linear correlation between genetic and geographic distances (dots in the black ellipse in Figure 4) would be far more pronounced. In other words, the distinctive feature of this subgroup is common long-distance transfer of viruses (or infected ticks) by an unknown animal with area restricted to Sarmatic mixed forest, Scandinavian and Russian taiga, and Urals montane tundra and taiga. A much more speculative assumption would be that replication is possible directly in an unknown flying animal. There are two groups of vertebrate animals that can fly with ticks: bats [47,48,49] and birds [50,51,52,53,54,55]. The role of bats in the distribution of TBEV is currently unknown. This may be due to an absence of studies where samples from bats were tested for TBEV antibodies. To the best of our knowledge, such studies were conducted over 40 years ago (https://pubmed.ncbi.nlm.nih.gov/13502551/; https://pubmed.ncbi.nlm.nih.gov/683144/; https://www.cabdirect.org/cabdirect/abstract/19612702944) [56,57,58]. However, bat participation at different levels in the circulation of several other flaviviruses (Dengue, Yellow fever, West Nile, Zika, Usutu, St. Louis encephalitis, Kyasanur forest disease, Japanese encephalitis viruses) has been demonstrated [59]. Coupled with the fact that bats can carry ticks, their role in TBEV distribution cannot be excluded.

Ticks feeding on birds may be infected by TBEV [60,61]. Moreover, TBEV markers (viral RNA and antigen) [52] or antibodies against TBEV [62] may be found directly in birds. This means that birds may be potentially highly involved in TBEV transmission routes. Noteworthy, several passerine birds are “ring species” [63] consisting of reproductively isolated subspecies. For example, great tit (*Parus major*) complex is divided into four groups that could be considered as separate species [64]. These four subspecies are distributed in different geographical regions. The distribution area of another passerine, western greenish warbler (*Phylloscopus trochiloides viridanus*), a subspecies of *P. trochiloides*, is slightly larger than known Baltic TBEV Sib subgroup geography [65]. Nevertheless, to unambiguously confirm the assumption about the active participation of unknown flying animals in the circulation of the Baltic TBEV subgroup, extensive fieldwork is required.

## 5. Conclusions

The limited Baltic TBEV-Sib distribution area coincides with active mixing of viruses between distant locations. This may be a consequence of active TBEV dissemination by flying animals. Most possibly, this animal is a bird distributed in the Sarmatic mixed forest, Scandinavian and Russian taiga, and Urals montane tundra and taiga. TBEV dissemination may occur either indirectly, via infected tick migration, or directly, if the virus can replicate in a flying animal.
